# Hand dexterity and mobility independently predict cognition in older adults: a multi-domain regression analysis

**DOI:** 10.3389/fnagi.2025.1624307

**Published:** 2025-08-26

**Authors:** Thomas Rudolf Schneider, Ansgar Felbecker, Ben von Mitzlaff, Gregor Weissofner, Sarah Meier, Patrick Eggenberger, Simon Annaheim

**Affiliations:** ^1^Department of Neurology, HOCH Health Ostschweiz, Cantonal Hospital of St. Gallen, St. Gallen, Switzerland; ^2^Department of Neurology, University Hospital of Bern, Bern, Switzerland; ^3^Department of Neurology, Cantonal Hospital of Lucerne, Lucerne, Switzerland; ^4^Department of Medicine, Cantonal Hospital Münsterlingen, Münsterlingen, Switzerland; ^5^Empa, Swiss Federal Laboratories for Materials Science and Technology, Laboratory for Biomimetic Membranes and Textiles, St. Gallen, Switzerland; ^6^Department of Health Sciences and Technology, Institute of Human Movement Sciences and Sport, ETH Zurich, Zurich, Switzerland; ^7^Department of Health, OST Eastern Switzerland University of Applied Sciences, St. Gallen, Switzerland

**Keywords:** cognitive aging, motor function, multi-domain assessment, nine-hole peg test (NHPT), short physical performance battery (SPPB)

## Abstract

**Background:**

Motor function is a sensitive indicator of cognitive aging but the unique contributions of different motor domains are unclear when assessed together.

**Methods:**

We evaluated 98 community-dwelling older adults (median age: 74). From a neuropsychological battery, a primary Global Cognitive Composite score (GCCS) and three secondary domain scores were derived using Principal Component Analysis (PCA). Motor predictors included the Nine-Hole Peg Test (NHPT), grip strength, Apraxia Screen of TULIA (AST), SPPB sub-tests (5-chair-rises time (5CRT), 4 m-walk time (4MWT), balance), and inertial measurement unit (IMU)-based gait parameters. Stepwise regression controlling for age and sex identified robust predictors of the GCCS.

**Results:**

The final model identified several significant, independent motor predictors of the GCCS. Poorer hand dexterity (NHPT; *β* = −0.29, *p* < 0.01), slower 5CRT (*β* = −0.28, *p* < 0.01), and slower 4MWT (*β* = −0.17, *p* = 0.03) were associated with worse cognitive performance, while greater minimum toe clearance was associated with better performance (*β* = 0.19, *p* = 0.01). In contrast, grip strength, balance, usual gait speed, and measures of gait variability were not retained. The model explained 50.3% of the variance (Adjusted R^2^) in global cognitive performance.

**Conclusion:**

Hand dexterity (NHPT) and specific functional mobility tasks (5CRT, 4MWT) are robust, independent predictors of cognition in older adults. Grip strength, balance, usual gait speed, and gait variability offer limited additional value when assessed together. The NHPT and timed SPPB components are accessible, pragmatic tools for motor-cognitive research and screening.

## Introduction

A growing body of research highlights strong links between motor functioning and cognitive health in community-dwelling older adults (Fried et al., 2001; Buchman et al., 2007; Montero-Odasso et al., 2012; Wang et al., 2025). The relationship between motor and cognitive functions in aging is a complex, dynamic, and reciprocal system in which motor and cognitive functioning usually decline in parallel (Zhao et al., 2021; van der Willik et al., 2021) while a decline in specific motor functions, like balance and fine motor control, may precede a decline in cognitive processing speed (Finkel et al., 2016).

While cognitive screening tools remain the gold standard for detecting early cognitive impairment, there is increasing recognition that motor function measures—particularly grip strength, hand dexterity, balance and gait performance—may serve as early physical indicators of cognitive decline and risk of imminent dementia (Buchman et al., 2007; Buchman and Bennett, 2011; Boyle et al., 2010; Boyle et al., 2009; McGrath et al., 2019). However, despite numerous studies linking individual motor parameters to cognition, few have examined these relationships in an integrated, multimodal framework (Malmstrom et al., 2005; Sverdrup et al., 2021; Doi et al., 2019).

Grip strength, widely used as a marker of overall muscle function (Fried et al., 2001), has been consistently associated with global cognition and specific domains such as processing speed and memory in community-dwelling seniors with normal and impaired cognition (Sverdrup et al., 2021; McGrath et al., 2019; Wang et al., 2023; Yang et al., 2022). Longitudinal analyses and meta-analyses show that lower grip strength in older adulthood predicts faster cognitive decline and higher dementia incidence (Buchman et al., 2007; Wang et al., 2025; Boyle et al., 2010; Doi et al., 2019; Duchowny et al., 2022; Jeong et al., 2018; Jeong and Kim, 2018; Sibbett et al., 2018; Kobayashi-Cuya et al., 2018; Samper-Ternent et al., 2008; Cui et al., 2021; Wu et al., 2023). However, these associations may not hold when grip strength is considered alongside other physical abilities, such as balance and gait (Veronese et al., 2016). Moreover, grip strength does not capture fine motor control, which may be more robustly linked to cognitive function than grip strength (Curreri et al., 2018; Zhang et al., 2024; Kobayashi-Cuya et al., 2018). Fewer studies have focused on hand dexterity (e.g., pegboard or finger-tapping tests) in relation to cognition, but emerging evidence points toward robust associations. Dexterity was associated with global cognition and cognitive domains such as processing speed and executive function in community-dwelling adults (Ashendorf et al., 2009) and clinical populations, including patients with multiple sclerosis (Abraham et al., 2024; Yozbatiran et al., 2006), Parkinson’s disease (Bezdicek et al., 2014), mild cognitive impairment and dementia (de Paula et al., 1999). Errors in the Grooved pegboard test (GPT) were associated with executive dysfunction in veterans (Tolle et al., 2020). Moreover, in a healthy aging cohort, performance on the Grooved Pegboard Test (GPT) was associated with both visuomotor tracking ability and the structural integrity of widespread brain networks, including frontal and parietal white matter tracts (Yao et al., 2020).

Despite these findings, hand dexterity remains an underutilized measure in cognitive aging research. A decline in manual dexterity has been linked to global cognitive decline in aging cohorts (Wang et al., 2023), with neuroimaging findings indicating that poorer fine motor performance relates to brain structural changes commonly seen in cognitive aging.

Limb apraxia, a neurological disorder characterized by an impaired ability to perform learned skilled movements, such as tool use, is commonly observed in various dementia subtypes. Its highest prevalence has been reported in Alzheimer’s disease and frontotemporal dementias, though it also occurs in a subset of individuals with mild cognitive impairment (MCI) (Baumard et al., 2018; Baumard et al., 2016; Smits et al., 2014; Ozkan et al., 2013; Lesourd et al., 2013). Despite its potential as an early marker of cognitive impairment, the applicability of apraxia screening tools, such as the Apraxia Screen of TULIA (AST) (Vanbellingen et al., 2011), in community-dwelling older adults remains largely unexplored.

Slower gait speed and poorer lower-body function have repeatedly been associated with lower cognitive test performance and greater risk of cognitive decline (Handing et al., 2020; Collyer et al., 2022). For example, a pooled analysis of 17 studies found that better global cognition (via Mini Mental State Examination, MMSE or Montreal Cognitive Assessment, MoCA) correlates with faster usual gait speed, better balance, and quicker chair stands on the Short Physical Performance Battery (SPPB) (Handing et al., 2020). Similarly, longitudinal cohort studies report that older adults with concurrent decline in walking speed and memory exhibit substantially elevated dementia risk (Collyer et al., 2022). In addition to gait speed, spatial (e.g., stride length) and temporal (e.g., cadence, double support time) gait parameters as well as a greater intraindividual variability in stride length, swing time, and stance time have been linked to cognitive decline, with abnormalities in gait patterns preceding cognitive decline in some cases by several years (Montero-Odasso et al., 2012; Beauchet et al., 2018; Beauchet et al., 2016; Allali et al., 2016; Montero-Odasso et al., 2018; Savica et al., 2017). The cognitive control of gait, particularly in dual-task walking, is thought to depend on executive function and attention, making gait analysis a promising tool for early cognitive screening (Montero-Odasso et al., 2012).

Despite the well-documented associations between motor functioning and cognition, most previous studies have examined grip strength, dexterity, balance, and gait separately. This leaves a gap in understanding their independent contributions to cognitive function when considered together in a multimodal framework. Furthermore, it remains unclear whether combining upper limb motor measures with lower limb function (balance and gait) improves the prediction of cognitive performance.

This analysis is a substudy of a larger project aimed at developing a non-invasive, wearable system for the early prediction of cognitive decline focusing on the relationship between clinical motor assessments and cognitive performance.

Here, we applied a multidomain regression framework to isolate the most robust motor indicators of cognitive health in community-dwelling seniors. Our primary aim was to identify which motor measures were most strongly associated with a data-driven Global Cognitive Composite score. As a secondary aim, to uncover more nuanced relationships, we explored the links between motor performance and distinct, empirically derived cognitive domains.

## Methods

### Study population

Community-dwelling older adults (>65 years) were recruited via public advertisements, local senior organizations, and senior residence facilities. In parallel, patients with a Mild Cognitive Impairment (MCI) diagnosis were informed about the study at the Geriatric and Neurologic memory clinics in St. Gallen. Inclusion criteria required participants to be able to walk at least 5 min without rest (with or without a walking aid) and have sufficient hearing and vision (with correction if necessary). The main exclusion criteria were a history of dementia, stroke, other neurological conditions known to significantly affect motor function (e.g., Parkinson’s disease), severe psychiatric disorders, or acute/unstable chronic diseases. Measurements took place from November 2020 to March 2021. Informed consent was obtained from all participants. The study procedures were approved by the local ethics committee of Eastern Switzerland (Project ID 2020–00558) and conducted in accordance with the Declaration of Helsinki.

### Study procedures

This cross-sectional sub study uses data from a larger project aimed at developing a non-invasive, multi-parameter system for the early prediction of cognitive decline.

### Lower-limb function assessment

Participants underwent a series of assessments in a single session. Gait parameters were measured using a single-task walking protocol, which consisted of walking back and forth four times on a 20-m track at the individual’s preferred comfortable pace. Gait was recorded using two inertial measurement unit (IMU) sensors (Physilog 5, GaitUp, Switzerland), a system demonstrated to have good to excellent validity and test–retest reliability for spatiotemporal gait parameters when compared to a gold-standard optical motion capture system (Lefeber et al., 2019). For consistent placement, sensors were securely fastened to the top of each participant’s shoes with a strap. We relied on the manufacturer’s factory calibration for all sensors. After the gait task, lower-body function was assessed using the three disaggregated components of the Short Physical Performance Battery (SPPB) (Guralnik et al., 1994), which comprises balance tests, a 4-m walk test, and a 5-chair-rises test (5CRT). To create a more sensitive measure of balance and mitigate potential ceiling effects, an extended version of the standard balance test was administered. Participants first completed the standard SPPB balance protocol; those who achieved the maximum score of 4 points (by holding a tandem stand for 10 s) subsequently completed a more challenging extended balance test (Eggenberger et al., 2015). This extended test included a 20-s single-leg stance with eyes open (1 point for reaching 10 s, an additional point for 20 s) and a timed single-leg stance with eyes closed (1 point awarded for every 5 s maintained). The scores from both the standard and extended tests were then combined into a single, more granular balance score for the analysis. For the other two components, the continuous time (in seconds) taken to complete the 5-chair-rises test and the 4-meter walk test were used for further analysis.

### Upper extremity function assessment

Maximal isometric grip strength was measured using a Jamar dynamometer (JLW Instruments, USA). Adhering to a standardized protocol (Therapists et al., 2015), participants were seated with their shoulder adducted, elbow flexed at 90°, and forearm in a neutral position, with the examiner supporting the base of the instrument. Following a demonstration, they performed three trials alternating between hands, with a 60-s rest between attempts, while receiving standardized verbal encouragement. Fine motor dexterity was assessed with the Nine-Hole Peg Test (NHPT) (Mathiowetz et al., 1985). Following a single, non-timed practice trial, participants completed two timed trials with each hand. The fastest of the two trials was used for analysis. To account for accuracy, the timer continued to run if a participant dropped a peg. If a peg was dropped out of the participant’s reach, the examiner returned it to the bowl to allow the trial to continue. Limb praxis ability was screened using the Apraxia Screen of TULIA (AST) (Vanbellingen et al., 2011).

### Neuropsychological assessments

Cognitive performance was evaluated across multiple domains. Global cognition was assessed with the Quick Mild Cognitive Impairment screen (QMCI) (O'Caoimh et al., 2012). Episodic memory was measured using the Face-Name Associative Memory Exam (FNAME-12) (Papp et al., 2014). Executive function and processing speed were assessed with the Stroop Color-Word Test (parts A, B and C) and the Trail Making Test (TMT) parts A and B. Specifically, Stroop A and TMT-A provided indices of processing speed, while the Stroop interference conditions B and C and TMT-B assessed executive functioning. Verbal fluency was tested with both semantic fluency (e.g., naming animals or supermarket items) and phonemic fluency (letters F and A) tasks (Mueller et al., 2015; Clark et al., 2009).

### Data processing

For grip strength, we used the maximum force achieved in any of three trials by either hand as the representative score. To facilitate cross-study comparisons and account for demographic differences, this value was then Z-transformed using published age- and sex-specific normative data from a large pooled analysis of 12 British cohort studies (Dodds et al., 2014). NHPT performance was quantified as the mean of the fastest completion time from each hand (lower times indicate better dexterity). AST performance was recorded as the lower (more errors) score out of the two hands.

Gait data from the 4 × 20 m walk was processed with Gait Analyser software (v1.1.0, GaitUp, Switzerland). Two gait cycles around each turnaround point were removed to avoid turn-related variability. We extracted averaged values for gait variables previously associated with cognitive decline (Savica et al., 2017), including: speed [m/s], mean swing phase duration [% of stride], minimal toe clearance [m] during swing, and gait variability measures (coefficients of variation [CV] for step length and for swing duration).

Classification between minor cognitive impairment (MCI) and normal cognition was based on the Quick Mild Cognitive Impairment (QMCI) screen, using previously established cut-off scores that are stratified by age and years of education (yoe). Specifically, a participant was classified as impaired if their score was: <65 (for age ≤75 years and <12 yoe), <69 (for age ≤75 years and ≥12 yoe), <64 (for age >75 years and <12 yoe), or <70 (for age >75 years and ≥12 yoe) (O’Caoimh et al., 2017).

Data processing was conducted in R using the “tidymodels,” “mice,” and “bestNormalize” packages (Kuhn et al., 2025; van Buuren and Groothuis-Oudshoorn, 2011). Missing values exceeding 5% of participants were imputed using multiple imputation by chained equations (mice) with predictive mean matching, while those with <5% missingness were imputed using median values. All numeric predictors and outcomes were normalized using ordered quantile normalization followed by z-score standardization.

### Statistical analyses

Statistical analyses were performed in the R environment for statistical computing [version 4.4.2 (R Core Team, 2023)]. We used the “gtsummary” package for descriptive tables and exploratory age group comparisons. Categorical variables are presented as counts with percentages (%), and continuous variables as medians with interquartile ranges (IQR). Group comparisons between cognitive groups were conducted using the Kruskal-Wallis rank-sum test (continuous variables) and the Pearson *χ*^2^-test (categorical variables).

#### Principal component analysis of cognitive outcome measures

We performed a Principal Component Analysis (PCA) on the 10 individual normalized neuropsychological test scores as a data-driven approach to derive a Global Cognitive Composite score as well as cognitive domains from the covariance structure of the data itself. Prior to the PCA, raw scores for timed tests (Stroop A-C, TMT-A/B) were inverted so that higher values uniformly indicated better performance. The QMCI score was excluded from PCA as it represents a composite score. The suitability of the data for PCA was confirmed using Bartlett’s Test of Sphericity (Bartlett, 1951), which was significant (*p* < 0.001), and the Kaiser-Meyer-Olkin (KMO) measure of sampling adequacy (Kaiser and Rice, 1974). The overall Measure of Sampling Adequacy (MSA) was 0.72, and individual MSA values for all items were above 0.74, indicating that the data were appropriate for factor analysis (see also Supplementary Figure S1).

1. The primary outcome was a Global Cognitive Composite, representing the unrotated first principal component (PC1) from a PCA including all 10 tests.
2. The secondary outcomes were Domain-Specific Composites, based on a three-component solution identified by the scree plot and Kaiser’s criterion (eigenvalues > 1, see results). Given that cognitive domains are theoretically related, an oblique (Promax) rotation was applied to achieve a more interpretable solution.

### Predictors

The initial set of predictors was grouped into the following modalities:

- Demographics: Age, sex, body mass index (BMI), years of education
- Upper limb function: grip strength, NHPT time, AST score
- Lower limb functioning and Gait: 4 m walk test time, 5-chair-rises test time, extended balance test score, gait speed over 80 m, swing duration, minimal toe clearance, CV step length, CV swing duration.

#### Regression modeling

To identify robust predictors of cognitive performance, we employed a two-stage regression analysis. This approach was designed to first select a parsimonious set of candidate predictors from each domain (demographics, upper limb function, and lower limb function) based on our primary outcome before fitting a final multi-domain model with all predictors selected from the domain-specific models.

**Figure 1:**
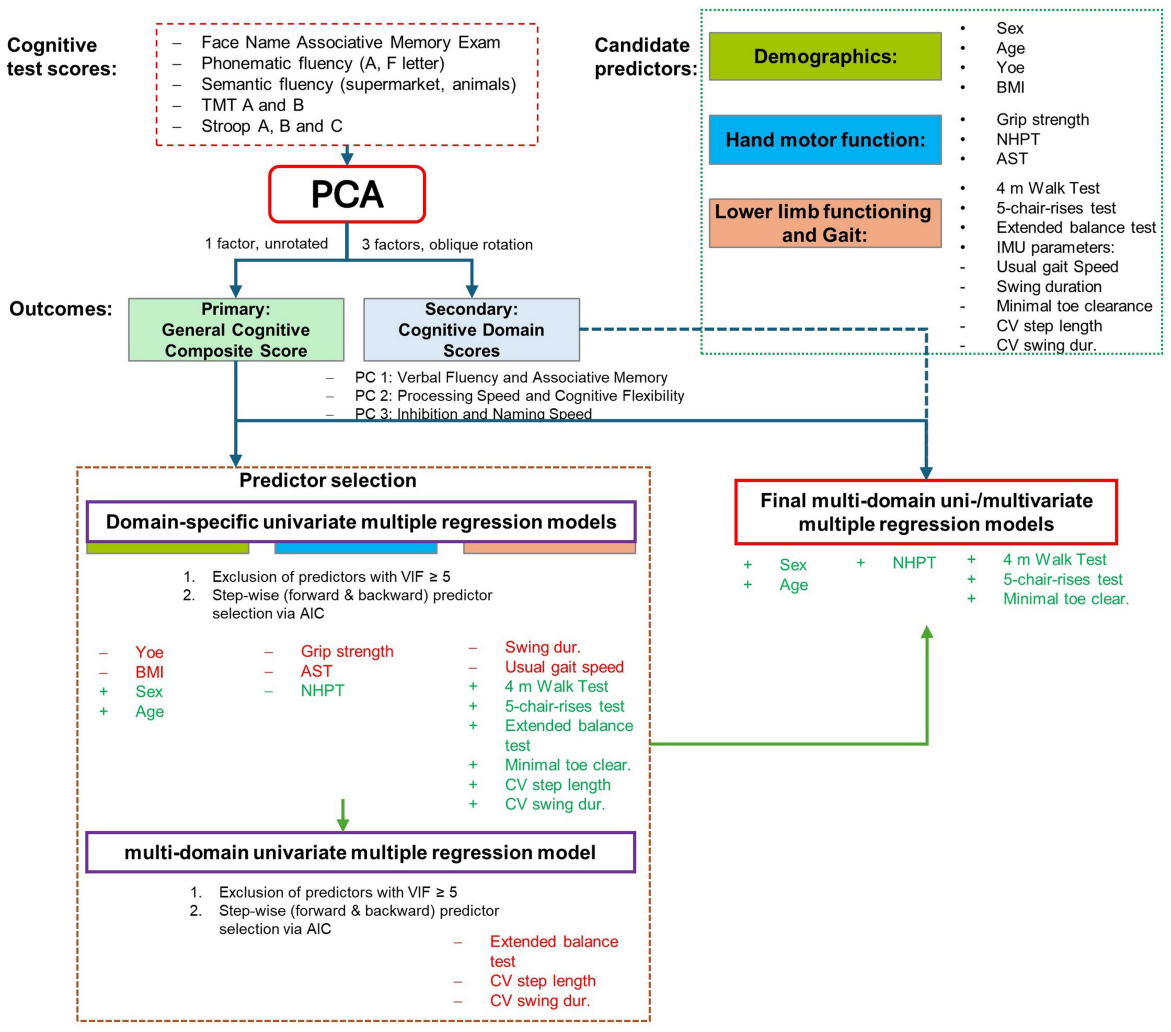
Overview of the statistical modeling approach. The figure illustrates the multi-stage analysis pipeline. First, data-driven cognitive outcomes—a primary global cognitive composite score and secondary cognitive domain scores—were derived from the individual neuropsychological tests using Principal Component Analysis (PCA). Next, a parsimonious set of predictors was selected by fitting domain-specific models to predict the primary outcome only, followed by stepwise AIC selection. Finally, this single set of predictors was used in two separate analyses: a multiple regression model predicting the primary global score, and a multivariate multiple regression model predicting the secondary domain scores. PCA, Principal Component Analysis; AIC, Akaike Information Criterion; TMT, Trail Making Test; FNAME, Face-Name Associative Memory Exam; NHPT, Nine-Hole Peg Test; AST, Apraxia Screen of TULIA; SPPB, Short Physical Performance Battery.

First, domain-specific models were fitted for each modality (demographics, upper limb function, and lower limb function) to predict our primary outcome, the Global Cognitive Composite score. Predictor collinearity was assessed via variance inflation factor (VIF), and predictors with VIF ≥ 5 were excluded. However, there were no collinearity issues indicated by a VIF ≥ 5. Within each of the domain-specific models, stepwise model selection (combining forward and backward steps) based on Akaike Information Criterion (AIC) was applied. Subsequently, all candidate predictors that were retained in the domain-specific models were entered into the final multi-domain regression model, again predicting the Global Cognitive Composite score. A final stepwise AIC selection was applied to this combined model to determine the most parsimonious set of predictors from across all domains. To explore more nuanced relationships with cognitive domains, a separate multivariate multiple regression model using the exact same set of predictors was fitted to jointly predict our secondary outcomes, the data-driven cognitive domain scores. This multivariate approach accounts for the shared variance among the cognitive domains, while minimizing model complexity and the risk of overfitting. To explore whether the associations between motor performance and cognition differed by sex, follow-up models adding sex-by-motor interaction terms to the final models predicting the global cognitive score and cognitive domains were fitted (Figure 1).

All statistical tests were two-tailed with the level of significance set at *α* ≤ 0.05. Regression results are reported as standardized beta coefficients (*β*) with associated *p*-values and 95% confidence intervals. The models’ assumptions were verified through comprehensive diagnostic checks using the performance package (Lüdecke et al., 2021)—including visual inspection of Q-Q plots (for normality) and residual-vs-fitted plots (for linearity and homoscedasticity), as well as formal tests for multivariate normality and influential cases using the mvinfluence package (Friendly, 2022).

## Results

### Participant characteristics

A total of 98 community-dwelling older adults (median age: 74 years; IQR: 70–79, 43 men and 55 women) participated in the study. Table 1 summarizes participant characteristics by cognitive status. Based on revised QMCI cut-offs, 74 participants (75.5%) were classified as cognitively normal (CN) and 24 (24.5%) as having mild cognitive impairment (MCI). Participants with MCI were older (78.5 vs. 74.0 years, *p* < 0.001) and more often male (33% female vs. 62%, *p* = 0.026). No differences were observed in education, BMI, diabetes, hypertension, walking aid use, or fall history (all *p* > 0.05).

**Table 1:** Participant characteristics stratified by cognitive group according to the QMCI test.

Characteristic	Overall *N* = 98	CN *N* = 74	MCI *N* = 24	*p*-value
Sex	54 (55%)	46 (62%)	8 (33%)	0.026
Age	74.0 (70.0, 79.0)	74.0 (69.0, 77.0)	78.5 (75.0, 82.0)	<0.001
Years of education	13.0 (13.0, 17.0)	13.0 (13.0, 17.0)	13.0 (13.0, 15.0)	>0.9
BMI	25.1 (23.2, 27.4)	25.1 (23.0, 27.4)	25.2 (23.5, 27.5)	0.7
Diabetes	9 (9%)	4 (5%)	5 (21%)	0.062
Hypertension	44 (45%)	30 (41%)	14 (58%)	0.2
Walking aid	2 (2%)	0 (0%)	2 (8%)	0.093
Falls within last year	16 (16%)	11 (15%)	5 (21%)	0.7
QMCI total	78 (69, 84)	80 (76, 86)	60 (56, 67)	<0.001
Face name test total	61 (43, 74)	65 (53, 78)	36 (12, 55)	<0.001
(Missing)	2	1	1	
TMT-A [s]	29.3 (24.5, 36.9)	27.1 (22.7, 35.4)	36.4 (29.0, 38.5)	0.001
TMT-B [s]	70.2 (53.4, 95.8)	66.7 (51.4, 81.4)	92.7 (69.2, 115.5)	0.010
Stroop A [s]	15.3 (14.0, 17.0)	15.1 (13.9, 16.7)	15.8 (14.7, 17.1)	0.2
Stroop B [s]	22.2 (19.8, 25.1)	22.0 (19.3, 24.7)	23.2 (22.1, 27.1)	0.047
Stroop C [s]	39.6 (34.8, 46.2)	38.3 (33.8, 42.8)	42.3 (38.6, 54.8)	0.004
Letter fluency: F [correct items]	17 (12, 20)	17 (14, 21)	14 (9, 16)	0.001
Letter fluency: A [correct items]	16 (13, 19)	17 (14, 21)	13 (10, 15)	<0.001
Category fluency: supermarket [correct items]	18 (13, 25)	22 (14, 28)	17 (10, 19)	0.004
Category fluency: animals [correct items]	25 (17, 30)	26 (22, 31)	16 (13, 21)	<0.001
SPPB score	12 (12, 12)	12 (12, 12)	12 (10, 12)	0.044
(Missing)	1	1	0	
Extended balance score	6 (5, 7)	6 (5, 7)	6 (4, 6)	0.034
4 m walk test [s]	3.1 (2.8, 3.4)	3.1 (2.8, 3.4)	3.1 (2.8, 3.5)	0.7
5-chair-rises test [s]	9.3 (8.2, 10.8)	9.2 (8.1, 10.1)	10.3 (8.9, 11.5)	0.034
(Missing)	1	1	0	
Swing (% of cycle dur.)	38.9 (38.1, 39.9)	38.8 (38.1, 39.7)	39.2 (38.2, 40.4)	0.4
(Missing)	4	2	2	
Usual gait speed (m/s)	1.4 (1.2, 1.5)	1.4 (1.2, 1.5)	1.3 (1.2, 1.5)	0.4
(Missing)	4	2	2	
Min toe clearance (cm)	2.2 (1.6, 2.8)	2.2 (1.7, 2.8)	1.9 (1.3, 3.0)	0.2
(Missing)	5	3	2	
CV Swing (%)	3.6 (2.8, 4.5)	3.4 (2.5, 4.4)	3.9 (3.3, 4.9)	0.045
(Missing)	4	2	2	
CV Step length (%)	5.1 (4.2, 5.8)	4.9 (4.0, 5.5)	5.8 (5.1, 6.4)	0.001
(Missing)	4	2	2	
max hand grip strength (Z-scored by normative data)	0.5 (−0.2, 1.1)	0.5 (0.0, 1.3)	0.1 (−0.3, 0.7)	0.14
(Missing)	1	1	0	
lower AST	12 (11, 12)	12 (11, 12)	11 (10, 12)	0.071
mean 9-HPT time	22.7 (20.9, 24.6)	21.8 (20.4, 23.8)	24.4 (23.0, 26.8)	<0.001

Numerical values are presented as medians with interquartile ranges (IQR), categorical values as frequencies and proportions (%). *p*-values indicate differences between cognitive groups (Wilcoxon rank-sum test for continuous variables, *χ*^2^ test for categorical variables). CN, Cognitively Normal; MCI, Mild Cognitive Impairment; BMI, Body Mass Index; QMCI, Quick Mild Cognitive Impairment screen; FNAME, Face-Name Associative Memory Exam; TMT-A/B, Trail Making Test Part A/B; AST, Apraxia Screen of TULIA; 9-HPT, Nine-Hole Peg Test; SPPB, Short Physical Performance Battery; CV, Coefficient of Variation.

Cognitive scores were lower in the MCI group: QMCI (60 vs. 80, *p* < 0.001), Face Name Test (36 vs. 65, *p* < 0.001), TMT-A (36.4 vs. 27.1 s, *p* = 0.001), TMT-B (92.7 vs. 66.7 s, *p* = 0.010), Stroop B (23.2 vs. 22.0 s, *p* = 0.047), and Stroop C (42.3 vs. 38.3 s, *p* = 0.004). Phonemic and semantic verbal fluency was also reduced: F words (13 vs. 17.0, p = 0.001), A words (13 vs. 17.0, *p* < 0.001), supermarket (17 vs. 22, p = 0.004), and animals (16 vs. 26, *p* < 0.001).

We found a small deterioration in lower limb function in MCI: SPPB (12 vs. 12, *p* = 0.044), extended balance score (6 vs. 6, *p* = 0.034), and 5CRT (10.3 vs. 9.2 s, p = 0.034). Gait variability was higher: CV swing (3.9% vs. 3.4%, *p* = 0.045) and CV step length (5.8% vs. 4.9%, p = 0.001). Other lower extremity parameters did not differ (all p > 0.05). Dexterity was reduced in MCI, as indicated by slower 9-HPT performance (24.4 vs. 21.8 s, *p* < 0.001), while AST and grip strength z-scores did not differ (both p > 0.05).

Supplementary Table S1 shows participant characteristics by sex. No sex differences were found in age, years of education, or BMI. Men showed a higher proportion of MCI (36% vs. 15%, p = 0.026) and performed worse on most cognitive tests, including QMCI (72 vs. 80, *p* < 0.001), Face Name Test (53 vs. 69, *p* < 0.001), and Stroop B (23.3 vs. 21.5 s, *p* = 0.002). Women scored lower on the SPPB (12 vs. 12, *p* = 0.017), but showed better dexterity (9-HPT: 21.6 vs. 23.4 s, *p* = 0.003) and higher grip strength z-scores (0.7 vs. 0.1, *p* = 0.005).

### PCA of neuropsychological tests

To derive empirical cognitive composites with minimal intercorrelation, we performed a Principal Component Analysis (PCA) on 10 normalized neuropsychological test scores. A three-component solution was selected based on the convergence of Kaiser’s Criterion (eigenvalues > 1) and a visual inspection of the Scree Plot’s elbow (Supplementary Figure S1). We then extracted two sets of scores.

First, a Global Cognitive Composite was created from the unrotated first principal component. This component explained 39.9% of the total variance, with all 10 tests showing strong positive loadings (range: 0.50–0.74; Supplementary Table S2).

Second, a more granular analysis using an oblique (Promax) rotation yielded three distinct cognitive domains, explaining a cumulative 63.9% of the variance (Supplementary Table S3). These domains were:

- PC 1: verbal fluency and associative memory: defined by strong loadings from category fluency (animals: 0.96; supermarket: 0.78), letter fluency (A: 0.63; F: 0.54), and the Face Name Test (0.56).
- PC 2: executive speed: defined by high loadings from the trail making Test B (0.95) and A (0.85).
- PC 3: inhibition and naming speed: defined by high loadings from the Stroop tests (Stroop B: 0.87; Stroop A: 0.84; Stroop C: 0.59).

The factor scores from the Global Cognitive Composite were used as the primary outcome, and the three domain-specific component scores were used as secondary outcomes in subsequent regression analyses.

### Regression results

#### Predictor selection using domain-specific models

To identify candidate variables for the final analysis, we first fitted separate regression models for each predictor domain (demographics, upper limb, and lower limb) on our primary outcome, the global cognitive composite score.

- Demographics: after stepwise selection, age (*β* = −0.40, *p* < 0.001) and female sex (*β* = 0.60, *p* < 0.001) were retained as significant predictors. This model explained 25.2% of the variance (Adjusted R^2^ = 0.252; Supplementary Table S4).
- Upper limb function: only nine-hole peg Test (NHPT) time was retained, showing a significant negative association with global cognition (*β* = −0.54, *p* < 0.001). This model explained 28.7% of the variance (Adjusted R^2^ = 0.287; Supplementary Table S5).
- Lower limb function and gait: stepwise selection retained several predictors. Significant associations were found for 5-chair-rises time (*β* = −0.22, *p* = 0.03), minimal toe clearance (*β* = 0.24, *p* = 0.01), and step length variability (*β* = −0.19, *p* = 0.04). The extended balance score and 4-meter walk time were also retained by the selection procedure but were not statistically significant at this stage (Supplementary Table S6).

#### Final multi-domain models

Subsequently, all predictors retained from the three domain-specific models were entered into a single regression model. A final stepwise selection was applied to this multimodal model to derive the most parsimonious set of predictors. In this final selection step, the extended balance score and measures of gait variability (CV step length, CV swing duration) were eliminated.

#### Primary outcome: global cognitive composite score

The final multimodal model significantly predicted the global cognitive composite score, explaining 50.3% of the variance (Adjusted R^2^ = 0.503; Table 2). In this model, older age (*β* = −0.23, *p* < 0.01) was associated with lower cognitive scores, while female sex was associated with higher scores (*β* = 0.45, *p* < 0.01). For motor performance, poorer hand dexterity (NHPT time: *β* = −0.29, *p* < 0.001) and slower lower-limb function on both the 5-chair-rises test (*β* = −0.28, *p* < 0.01) and the 4-meter walk test (*β* = −0.17, *p* = 0.03) were independently associated with worse global cognition. Conversely, greater minimal toe clearance was associated with better cognitive scores (*β* = 0.19, *p* = 0.01).

**Table 2:** Final regression model for multi-domain predictors of global cognitive composite scores after StepAIC forward and backward predictor selection.

Predictors	PC: global cognition			
	Estimates	CI	Statistic	*p*
(Intercept)	−0.25	−0.47 to −0.02	−2.20	0.03
Female sex	0.45	0.13–0.76	2.84	0.01
Age	−0.23	−0.38 to −0.07	−2.90	<0.01
mean 9-HPT time	−0.29	−0.46 to −0.13	−3.49	<0.01
4 m walk test [s]	−0.17	−0.33 to −0.01	−2.16	0.03
5-chair-rises test [s]	−0.28	−0.44 to −0.12	−3.41	<0.01
Min toe clearance (cm)	0.19	0.05–0.34	2.62	0.01
Observations	98			
R^2^/R^2^ adjusted	0.534/0.503			
AIC	218.302			

Residual df: 91.

Diagnostic checks confirmed that all key model assumptions were met, with no significant violations of linearity, normality of residuals, or homoscedasticity detected (Supplementary Figure S2). The independent associations of each predictor with the global cognitive score are illustrated in the partial effects plots in Supplementary Figure S3.

#### Secondary outcomes: cognitive domain scores

The final set of predictors was also used in a multivariate model to predict the three data-driven cognitive domain scores (see Table 3).

**Table 3:** Multivariate multiple regression results predicting cognitive domain scores.

Term	PC 1: Verbal fluency and associative memory	PC 2: Executive speed	PC 3: Inhibition and naming speed
(Intercept)	−0.31 [−0.57, −0.06]*	0.02 [−0.24, 0.28]	−0.24 [−0.51, 0.03]
Female sex	0.57 [0.21, 0.92]**	−0.03 [−0.40, 0.33]	0.43 [0.05, 0.81]*
Age	−0.15 [−0.33, 0.03]	−0.32 [−0.50, −0.14]***	−0.11 [−0.30, 0.08]
mean 9-HPT time	−0.22 [−0.41, −0.03]*	−0.30 [−0.50, −0.11]**	−0.20 [−0.41, −0.00]*
4 m walk test [s]	−0.13 [−0.31, 0.05]	−0.10 [−0.29, 0.08]	−0.20 [−0.39, −0.00]*
5-chair-rises test [s]	−0.28 [−0.47, −0.10]**	−0.14 [−0.32, 0.05]	−0.22 [−0.41, −0.02]*
Min toe clearance (cm)	0.16 [−0.00, 0.33]	0.19 [0.02, 0.36]*	0.11 [−0.07, 0.29]
Adjusted R^2^	**0.36**	**0.33**	**0.27**

The table displays standardized beta coefficients (*β*) with their corresponding 95% confidence intervals [95% CI] from the final multivariate model. The model uses the final selected predictor set indicated in bold to predict the three data-driven cognitive domain scores. The final row shows the Adjusted R^2^ value for each domain-specific model. Significance levels: *p < 0.05, **p < 0.01, ***p < 0.001.

For Verbal Fluency and Associative Memory (PC1), female sex (*β* = 0.57), slower NHPT performance (*β* = −0.22), and slower 5-chair-rises time (*β* = −0.28) were all significantly associated with lower scores.

For Executive Speed (PC2), older age (*β* = −0.32) and slower NHPT performance (*β* = −0.30) were associated with lower scores, while greater minimal toe clearance (*β* = 0.19) was associated with better scores.

Finally, for Inhibition and Naming Speed (PC3), female sex was associated with better performance (*β* = 0.43), whereas slower NHPT performance (*β* = −0.20), slower 4-meter walk time (*β* = −0.20), and slower 5-chair-rises time (*β* = −0.22) were all associated with lower scores.

The model explained 36, 33, and 27% of the variance in each of the three domains, respectively.

Diagnostic checks of the multivariate model confirmed that all key assumptions were met, with no significant violations of linearity, normality, or homoscedasticity, and no influential multivariate outliers detected (see Supplementary Figure S4).

#### Follow-up analysis of sex interactions

To explore whether the observed associations between motor performance and cognition differed by sex, we conducted follow-up analyses by adding sex-by-motor interaction terms to the final models for both the primary (global cognitive score) and secondary (cognitive domains) outcomes.

The results showed no significant interaction effects between sex and any of the motor predictors in either the model for the global score or in the multivariate model for the cognitive domains. The full results of this interaction model are presented in Supplementary Tables S7, S8.

## Discussion

In this study, we applied a rigorous multi-domain regression framework to identify which motor functions are the most salient independent predictors of cognition in older adults. The principal finding is that after controlling for demographic factors, poorer hand dexterity (NHPT), slower functional mobility (5-chair-rise and 4-m walk time), and reduced minimal toe clearance were all independently associated with lower global cognitive performance. Notably, hand dexterity emerged as a particularly robust indicator, predicting performance across all empirically derived cognitive domains. In contrast, other commonly used metrics such as grip strength, balance performance, usual gait speed, and gait variability were eliminated from the final models by AIC-based selection criteria, suggesting they offer limited additional predictive value when more specific motor measures are included. The final model, which also confirmed the significant roles of age and sex, explained approximately 50% of the variance in the global cognitive score, providing a strong benchmark for future research into motor-cognitive coupling.

Hand dexterity, as measured by the NHPT, emerged as the most robust and consistent motor predictor of cognition in our study. It was significantly associated not only with the global cognitive composite score but also with all three data-driven cognitive domains: verbal fluency and memory, executive speed, and inhibition. These results strongly support the idea that fine motor control is a particularly sensitive barometer of cognitive aging. The NHPT is more than a test of motor speed; its execution demands complex processes like visuomotor integration, sustained attention, and cognitive flexibility, which are known to be intertwined with cognitive health in older age (Ashendorf et al., 2009; de Paula et al., 1999). Our findings reinforce the NHPT’s utility as a simple and pragmatic measure for detecting subtle cognitive changes (Kobayashi-Cuya et al., 2018).

Furthermore, our findings align with neuroimaging work suggesting this strong motor-cognitive link is rooted in shared neural substrates. Performance on the grooved pegboard tasks has been associated with the structural integrity of frontoparietal white matter tracts—networks that are foundational to both sophisticated motor control and higher-order cognitive processing (Yao et al., 2020).

In contrast, grip strength—although commonly studied and associated with cognition in bivariate models (Buchman et al., 2007; Sibbett et al., 2018) —was not retained as predictor in the upper-extremity function specific model when NHPT and AST were included in the model. This finding supports the hypothesis that grip strength may function more as a general marker of sarcopenia or frailty, rather than a specific indicator of cognitive status, especially when assessed alongside more cognitively demanding motor tasks like the NHPT (Veronese et al., 2016; Kobayashi-Cuya et al., 2018).

In our analysis, the Apraxia Screen of TULIA (AST) was eliminated during the initial domain-specific variable selection process and therefore was not carried forward into the final multi-domain model. This early elimination indicates that, on its own, apraxia screening did not have sufficient predictive power for the global cognitive score compared to other motor variables. This is noteworthy because we descriptively observed that a notable proportion of participants achieved borderline scores, even while very few met the formal criteria for apraxia. While these borderline scores could hint at subtle praxis impairments, a cautious interpretation is warranted. The AST relies heavily on understanding verbal commands, so lower scores could be attributed to factors other than a true motor planning deficit. Potential confounds include difficulties with hearing or language comprehension, which may have been exacerbated by the mandatory use of face masks by both examiners and participants during the study period. Ultimately, the AST’s failure to be retained as a key predictor underscores its limited utility for assessing cognitive status in this cohort of community dwelling-seniors.

Prior research has firmly established strong associations between the composite Short Physical Performance Battery (SPPB), global cognition, and the risk of developing dementia (Wu et al., 2023; Veronese et al., 2016; Handing et al., 2020). However, a known limitation of the composite score, particularly in high-functioning cohorts like ours, is a potential ceiling effect which can obscure more nuanced relationships. To overcome this, our study employed a more granular analysis by disaggregating the SPPB, using the continuous time scores for the 5-chair-rises and 4-meter walk tests, and implementing an extended balance test involving challenging single-leg stances to create a more sensitive measure of postural control (Eggenberger et al., 2015). Our findings underscore the value of this granular approach. While slower times on the 5-chair-rises and 4-meter walk tests emerged as robust, independent predictors of lower global cognitive scores, the extended balance score did not. Notably, the balance measure was not even statistically significant in the initial domain-specific regression model, indicating its limited predictive power for cognition in this cohort relative to other measures of lower limb function and gait.

This dissociation between dynamic mobility and static balance is highly consistent with the primary conclusions of the recent systematic review and meta-analysis by Divandari et al. (2023), which concluded that the cognition-balance link is task-specific; dynamic balance tasks show a moderate association with cognition, while the link with static balance is small, likely because dynamic tasks are more cognitively demanding. Our results, therefore, support the view that tasks requiring the continuous integration of motor and cognitive processing are more salient indicators of cognitive status than postural control alone, reinforcing the clinical utility of these simple, timed mobility tests.

While slower gait speed is a recognized early indicator of cognitive decline, with motor slowing often preceding measurable cognitive deterioration (Collyer et al., 2022; Jayakody et al., 2022), our analysis revealed a key dissociation. We found that a short, timed 4-meter walk was a significant predictor of cognitive performance, whereas the average steady-state gait speed from a longer 80-meter walk was not. This distinction likely arises from the different cognitive demands of each task. The 4-meter walk is a goal-directed test requiring rapid initiation and control, phases heavily dependent on executive functions like motor planning and attention (Yogev-Seligmann et al., 2008). In contrast, the 80-meter assessment captured comfortable, steady-state walking—a more automatic process with a lower executive load (Clark, 2015), particularly as the cognitively demanding turning phases were removed from our analysis. Although numerous meta-analyses confirm that slower gait speed is a robust marker of cognitive impairment (Peel et al., 2019), this association can be less sensitive in higher-functioning cohorts, who may exhibit a “ceiling effect” on this measure (Windham et al., 2022). Similarly, other instrumented gait parameters expected to reflect cognitive function, such as gait variability—often regarded as a marker of cortical dysfunction (Pieruccini-Faria et al., 2021)—were not retained as independent predictors. The absence of this link may be explained by our use of a single-task assessment, as motor-cognitive links are often more robustly detected under challenging dual-task paradigms (Montero-Odasso et al., 2012). This result is consistent with the ambiguous findings in the literature regarding healthy older adults, where some studies link gait variability to executive dysfunction (Mukli et al., 2022), while others find no strong correlation in high-functioning populations (Valkanova et al., 2018). The one notable exception among our IMU-derived parameters was Minimal Toe Clearance (MTC), which was significantly associated with our Global Cognitive Composite and specifically with the executive speed domain. This finding aligns with evidence that MTC is highly sensitive to attentional and executive loading during walking and may represent an early motor marker of frontal-lobe dysfunction (Killeen et al., 2017), highlighting its potential value over more global measures like gait speed in a comprehensive assessment.

Demographic variables were significant contributors to cognitive performance in the final model. As expected, older age was independently associated with lower Global Cognitive Composite scores. Female sex also predicted better cognitive performance; however, this finding should be interpreted with caution as it may reflect sampling bias in our specific cohort rather than a generalizable finding. To address the more central question of whether the motor-cognition relationship itself differed by sex, we formally tested for interaction effects. This follow-up analysis revealed no significant interactions, suggesting that the fundamental associations between motor performance and cognition were consistent for both men and women in our sample.

Overall, our study highlights the specific associations of the Nine-Hole Peg Test alongside key functional mobility components of the SPPB with cognition in the elderly, supporting their continued use as simple, pragmatic tools for assessing motor-cognitive health in both research and clinical settings. Our findings are supported by recent longitudinal work from the Rush Memory and Aging Project, where a composite motor score including dexterity, gait, and grip strength was predictive of global cognitive decline and all tested domains over 5 years (Wang et al., 2023).

## Limitations

This study has several limitations:

First, its cross-sectional design precludes any conclusions about the directionality of the observed relationships.

Second, our study cohort was physically high functioning, which likely reflects a selection bias. Recruitment occurred during the COVID-19 pandemic, which may have deterred more frail individuals from participating and thus contributed to this bias. This is evidenced by the ceiling effect observed in the standard SPPB, which may have led to an underestimation of the true association between lower-limb function and cognition in a more heterogeneous older population.

Third, we assessed motor function exclusively under single-task conditions. While this isolates motor capacity, it is a limitation, as dual-task paradigms are often more sensitive for revealing subtle motor-cognitive interactions. Future research should explore these relationships under greater cognitive load.

Finally, while our multi-stage statistical approach was robust, the initial variable selection models were exploratory. Our primary, confirmatory conclusions are therefore drawn from the final multi-domain model that assesses the independent contribution of all retained predictors simultaneously.

## Conclusion

Our multi-domain regression analysis demonstrates that hand dexterity, measured by the NHPT, and specific functional mobility tasks—5-chair-rises time and 4-m walk time—are robust, independent predictors of cognitive performance in older adults. Notably, other commonly used measures, including grip strength, balance, and most instrumented gait metrics, did not provide independent predictive value in our comprehensive model. These findings underscore the importance of integrated modeling to disentangle the unique contributions of different motor domains to cognition. We conclude that simple, timed assessments like the NHPT and key SPPB components offer practical and accessible tools for cognitive screening and research, providing a valuable benchmark for evaluating emerging digital motor assessment technologies.

## Data Availability

The raw data supporting the conclusions of this article will be made available by the authors, without undue reservation.
