# Supplementary material for: Hand dexterity and mobility independently predict cognition in older adults: a multi-domain regression analysis
Source: Front Aging Neurosci. 2025 Aug 26;17:1624307. doi: 10.3389/fnagi.2025.1624307 (PMC12417501; doi:10.3389/fnagi.2025.1624307)
Supplement: Supplementary file 1 [file Data_Sheet_1.docx]

**SUPPLEMENTARY MATERIAL**

**Hand Dexterity and Mobility Independently Predict Cognition in Older Adults: A Multi-Domain Regression Analysis**

Thomas Rudolf Schneider, Ansgar Felbecker, Ben v. Mitzlaff, Gregor Weissofner, Sarah Meier, Patrick Eggenberger^°^ and Simon Annaheim°^*^

° Patrick Eggenberger and Simon Annaheim share senior authorship.


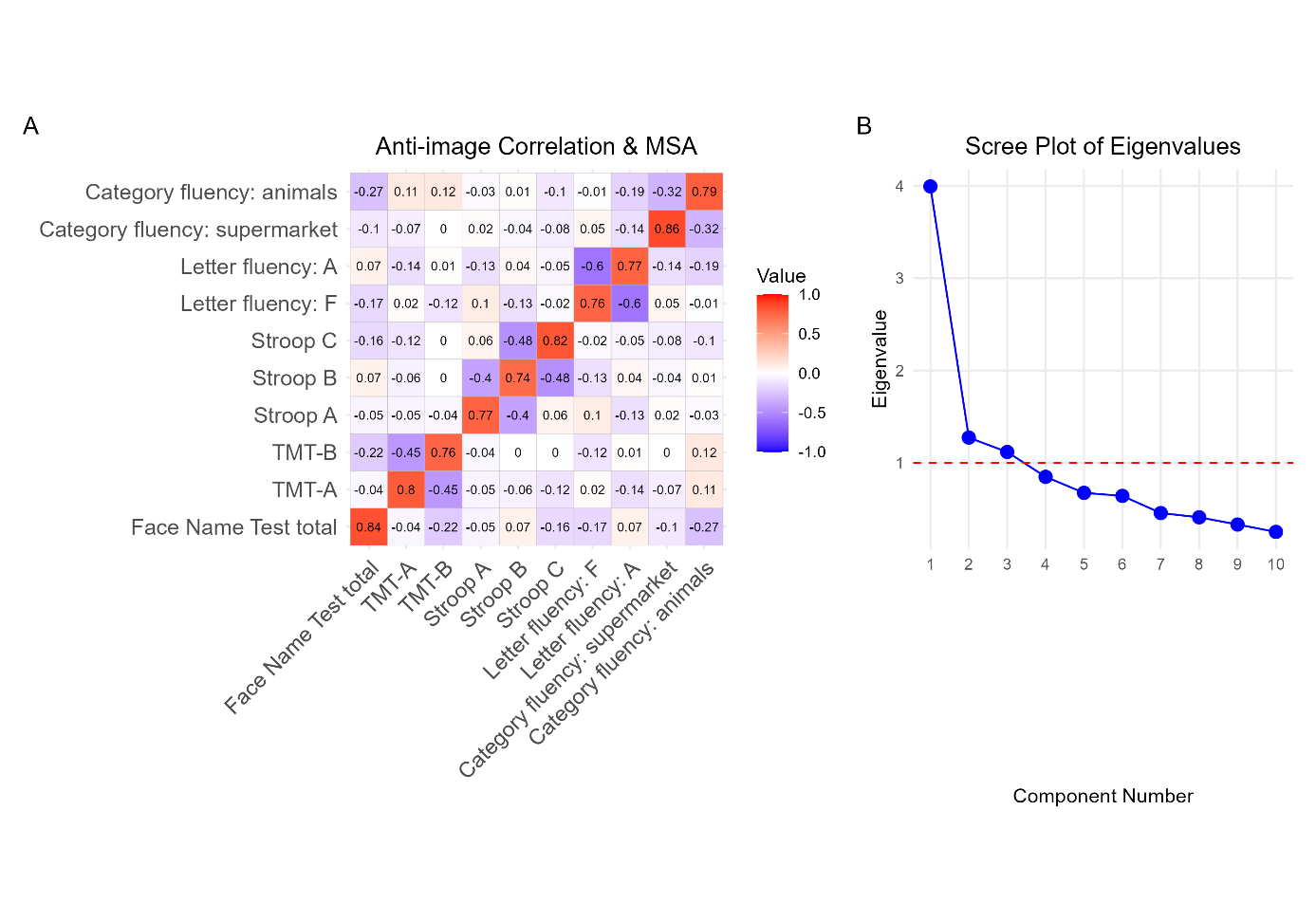


Supplementary Figure S 1: PCA Diagnostic Plots. Panel (A) shows the anti-image correlation matrix with Measure of Sampling Adequacy (MSA) on the diagonal. Panel (B) shows the scree plot of eigenvalues, where three components fall above the Kaiser criterion (dashed line), justifying their retention.


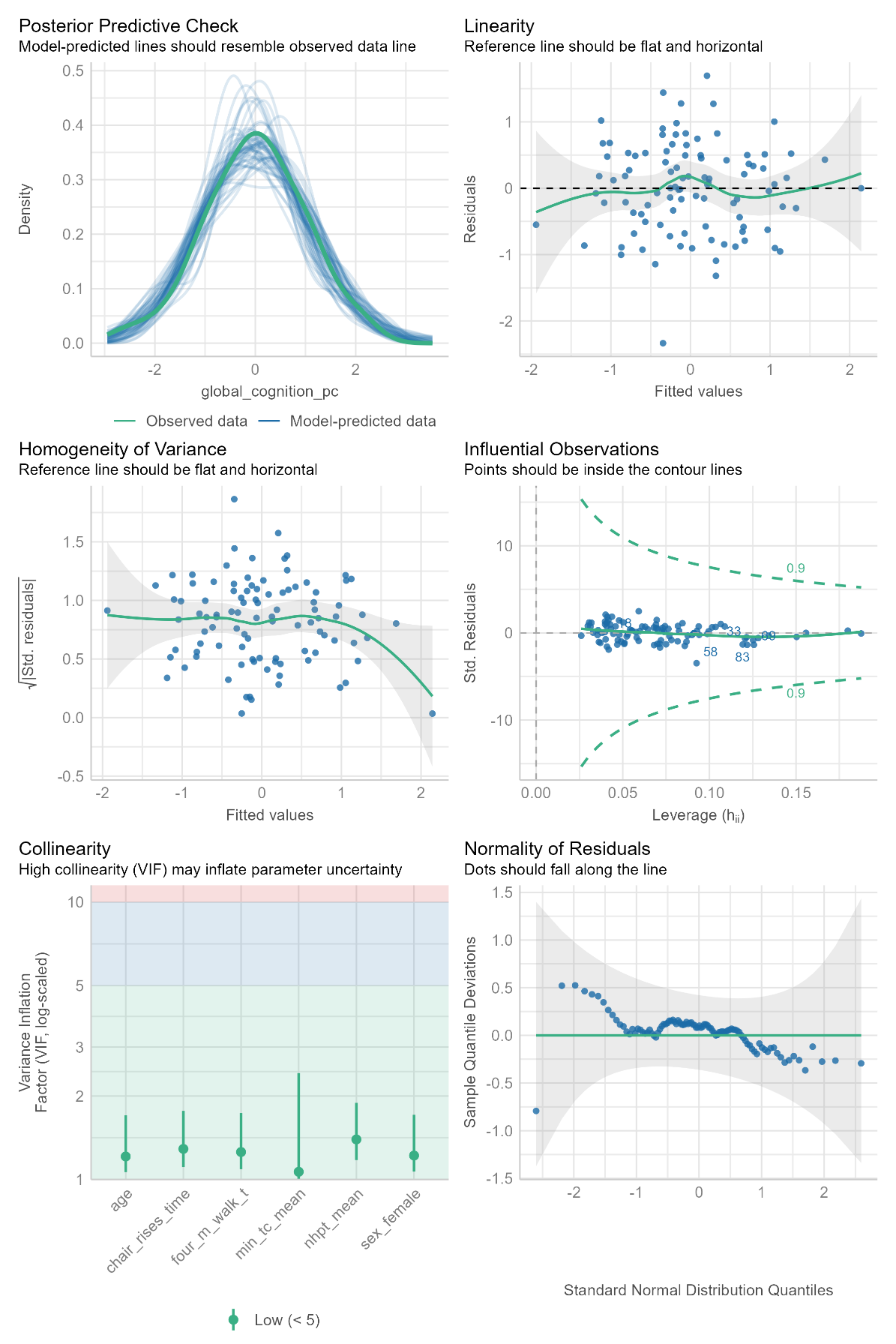


Supplementary Figure S 2: Diagnostic Plots for the Final Regression Model. Visual checks for key model assumptions. The plots display: residuals vs. fitted values to assess linearity and homoscedasticity; a Q-Q plot and density plot to assess the normality of residuals; Cook's distance to identify influential cases; and Variance Inflation Factors (VIF) to check for multicollinearity.


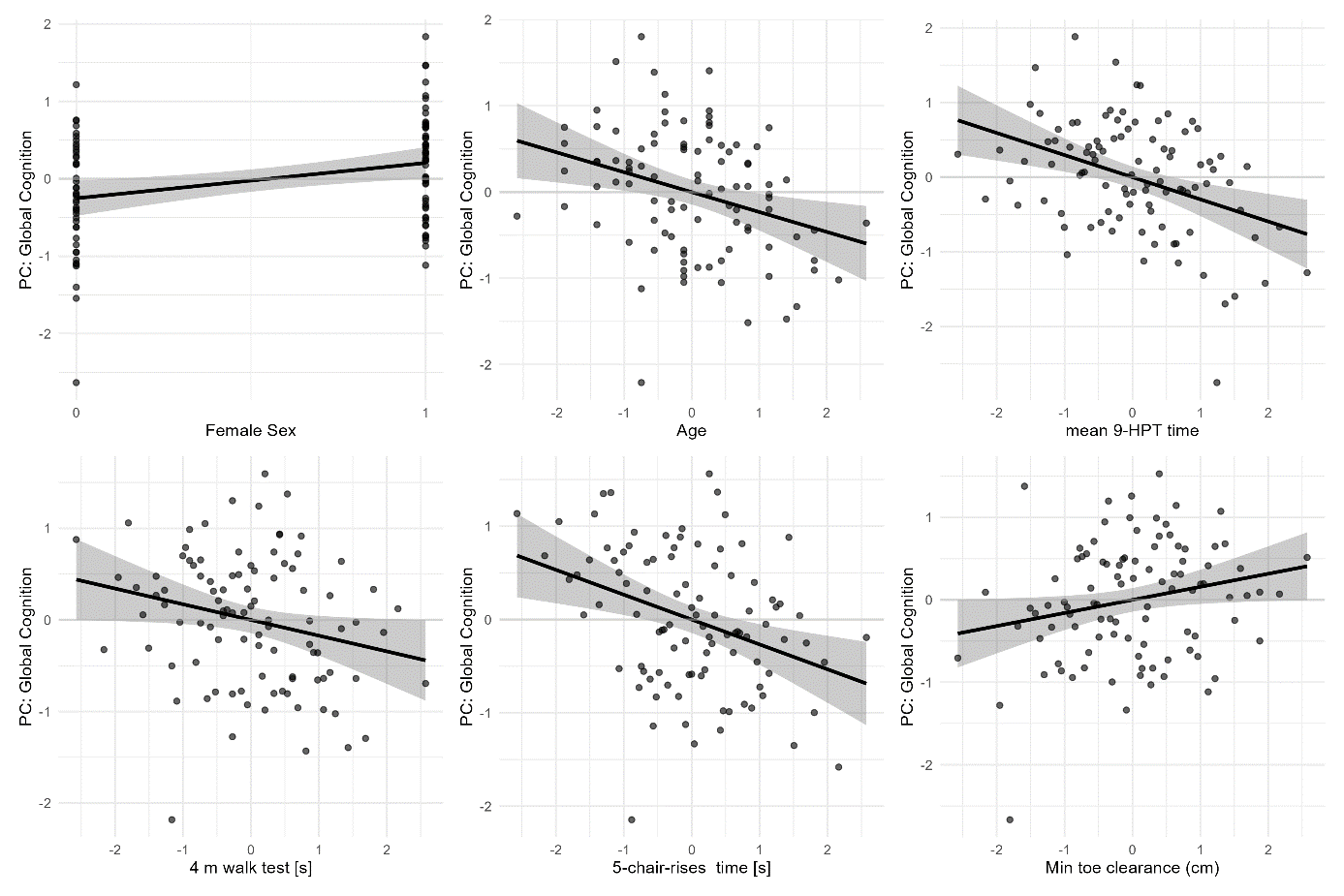


Supplementary Figure S 3: Partial Effects Plot for the Global Cognitive Score Model. Each panel shows the adjusted association between a Z-scored predictor (x-axis) and the global cognitive score (y-axis), controlling for all other model predictors. The points represent partial residuals, the solid line is the partial regression fit, and the shaded area is the 95% confidence interval.


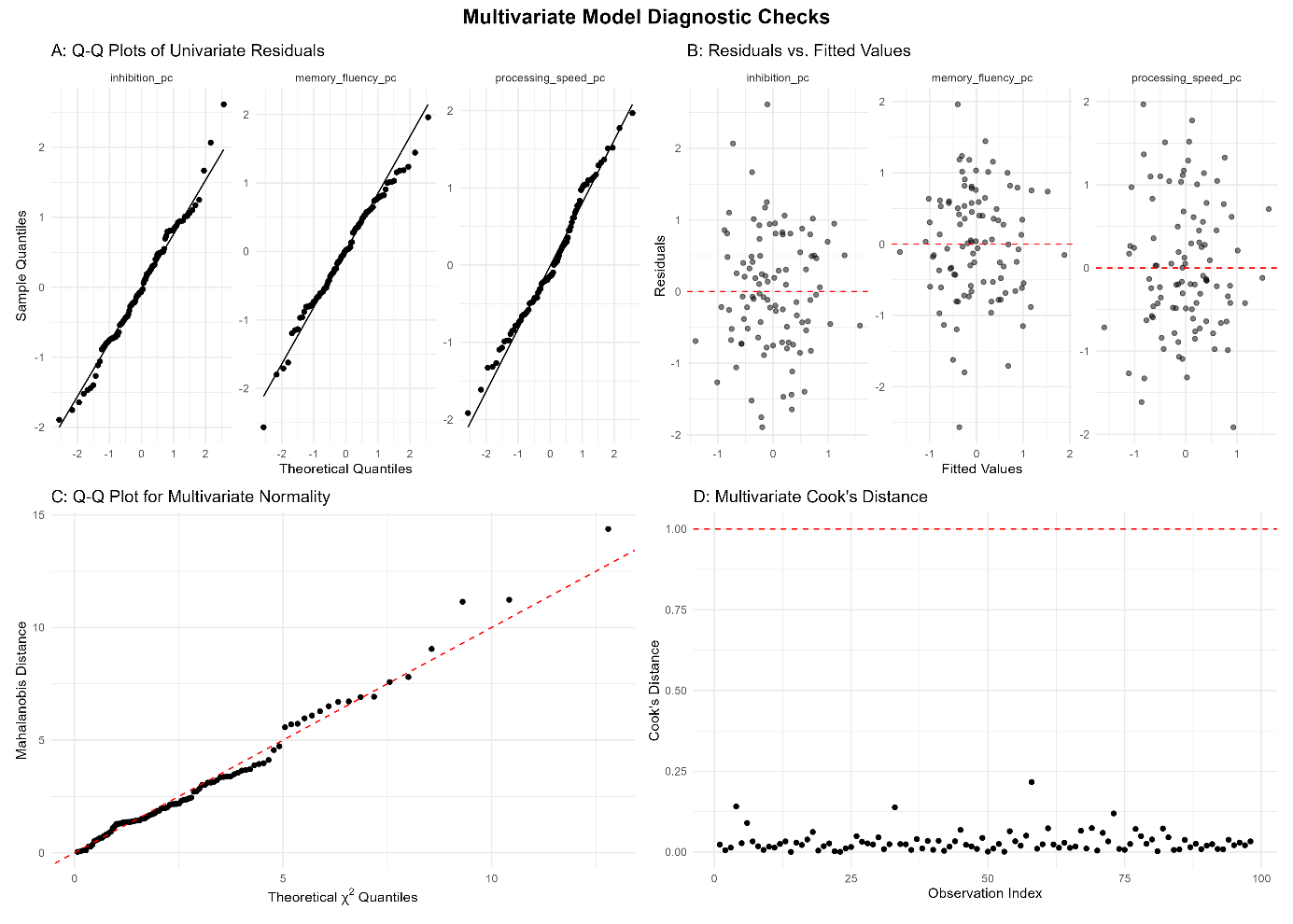


Supplementary Figure S 4: Diagnostic Plots for the Multivariate Model of Cognitive Domain Scores. The plots show visual checks for key statistical assumptions. (A) Q-Q plots assess the univariate normality of residuals for each outcome. (B) Residuals vs. fitted values plots assess linearity and homoscedasticity. (C) A Q-Q plot of Mahalanobis distances assesses multivariate normality of the full set of residuals. (D) An index plot of Cook's distance identifies influential cases.

Supplementary Table S 1: Participant characteristics stratified by sex. Numerical values are presented as medians with interquartile ranges (IQR), categorical values as frequencies and proportions (%). *p*-values indicate differences between men and women (Wilcoxon rank-sum test for continuous variables, χ² test for categorical variables).

| **Characteristic** | **Overall**  N = 98 | **male**  N = 44 | **female**  N = 54 | **p-value** |
| --- | --- | --- | --- | --- |
| **Cognitive group [QMCI]** | 24 (24%) | 16 (36%) | 8 (15%) | 0.026 |
| **Age** | 74.0 (70.0, 79.0) | 76.0 (70.0, 79.5) | 74.0 (71.0, 78.0) | 0.5 |
| **Years of education** | 13.0 (13.0, 17.0) | 13.0 (13.0, 17.0) | 13.0 (13.0, 13.0) | 0.3 |
| **BMI** | 25.1 (23.2, 27.4) | 25.2 (23.1, 27.1) | 25.1 (23.4, 27.6) | 0.9 |
| **Diabetes** | 9 (9%) | 7 (16%) | 2 (4%) | 0.084 |
| **Hypertension** | 44 (45%) | 20 (45%) | 24 (44%) | >0.9 |
| **Walking aid** | 2 (2%) | 1 (2%) | 1 (2%) | >0.9 |
| **Falls within last year** | 16 (16%) | 5 (11%) | 11 (20%) | 0.4 |
| **QMCI total** | 78 (69, 84) | 72 (60, 80) | 80 (74, 86) | <0.001 |
| **Face Name Test total** | 61 (43, 74) | 53 (35, 64) | 69 (55, 83) | <0.001 |
| (Missing) | 2 | 2 | 0 |  |
| **TMT-A [s]** | 29.3 (24.5, 36.9) | 29.6 (24.5, 36.9) | 29.2 (23.5, 36.7) | 0.9 |
| **TMT-B [s]** | 70.2 (53.4, 95.8) | 67.9 (52.4, 96.9) | 72.9 (54.4, 95.8) | 0.6 |
| **Stroop A [s]** | 15.3 (14.0, 17.0) | 15.8 (14.5, 17.1) | 14.9 (13.9, 16.6) | 0.2 |
| **Stroop B [s]** | 22.2 (19.8, 25.1) | 23.3 (21.9, 26.7) | 21.5 (19.1, 24.0) | 0.002 |
| **Stroop C [s]** | 39.6 (34.8, 46.2) | 41.1 (37.3, 51.0) | 37.9 (33.5, 42.8) | 0.013 |
| **Letter fluency: F [correct items]** | 17 (12, 20) | 15 (10, 19) | 17 (14, 22) | 0.011 |
| **Letter fluency: A [correct items]** | 16 (13, 19) | 16 (12, 18) | 16 (13, 21) | 0.15 |
| **Category fluency: supermarket [correct items]** | 18 (13, 25) | 17 (13, 25) | 21 (14, 28) | 0.045 |
| **Category fluency: animals [correct items]** | 25 (17, 30) | 21 (16, 30) | 25 (23, 30) | 0.052 |
| **SPPB score** | 12 (12, 12) | 12 (12, 12) | 12 (11, 12) | 0.017 |
| (Missing) | 1 | 0 | 1 |  |
| **Extended balance score** | 6 (5, 7) | 6 (6, 7) | 6 (4, 7) | 0.14 |
| **4 m walk test [s]** | 3.1 (2.8, 3.4) | 3.1 (2.8, 3.4) | 3.0 (2.8, 3.4) | 0.6 |
| **5-chair-rises time [s]** | 9.3 (8.2, 10.8) | 9.2 (8.2, 10.0) | 9.8 (8.2, 11.7) | 0.13 |
| (Missing) | 1 | 0 | 1 |  |
| **Swing (% of cycle dur.)** | 38.9 (38.1, 39.9) | 38.9 (38.1, 39.7) | 39.0 (38.1, 40.1) | 0.5 |
| (Missing) | 4 | 2 | 2 |  |
| **speed (m/s)** | 1.4 (1.2, 1.5) | 1.3 (1.2, 1.4) | 1.4 (1.2, 1.5) | 0.8 |
| (Missing) | 4 | 2 | 2 |  |
| **Min toe clearance (cm)** | 2.2 (1.6, 2.8) | 2.2 (1.4, 2.8) | 2.2 (1.8, 3.0) | 0.2 |
| (Missing) | 5 | 3 | 2 |  |
| **CV Swing (%)** | 3.6 (2.8, 4.5) | 3.6 (2.9, 4.2) | 3.5 (2.6, 4.7) | 0.7 |
| (Missing) | 4 | 2 | 2 |  |
| **CV Step length (%)** | 5.1 (4.2, 5.8) | 5.3 (4.6, 6.0) | 4.8 (4.1, 5.6) | 0.11 |
| (Missing) | 4 | 2 | 2 |  |
| **max hand grip strength (Z-scored by normative data)** | 0.5 (-0.2, 1.1) | 0.1 (-0.4, 0.9) | 0.7 (0.1, 1.7) | 0.005 |
| (Missing) | 1 | 0 | 1 |  |
| **lower AST** | 12 (11, 12) | 11 (11, 12) | 12 (11, 12) | 0.3 |
| **mean 9-HPT time** | 22.7 (20.9, 24.6) | 23.4 (21.8, 25.4) | 21.6 (20.1, 23.9) | 0.003 |
|  | | | | |
| Abbreviations: CN, Cognitively Normal; MCI, Mild Cognitive Impairment; BMI, Body Mass Index; QMCI, Quick Mild Cognitive Impairment screen; FNAME, Face-Name Associative Memory Exam; TMT-A/B, Trail Making Test Part A/B; AST, Apraxia Screen of TULIA; 9-HPT, Nine-Hole Peg Test; SPPB, Short Physical Performance Battery; CV, Coefficient of Variation. | | | | |

Supplementary Table S 2: Global Cognitive Composite Loadings. Component loadings of ten neuropsychological tests on the unrotated first principal component (PC1). All tests loaded positively on the component (range: 0.50-0.74), which explained 39.9% of the total variance

| Test | PC1 |
| --- | --- |
| Face Name Test total | 0.65 |
| TMT-A | 0.61 |
| TMT-B | 0.55 |
| Stroop A | 0.50 |
| Stroop B | 0.66 |
| Stroop C | 0.71 |
| Letter fluency: F | 0.71 |
| Letter fluency: A | 0.74 |
| Category fluency: supermarket | 0.59 |
| Category fluency: animals | 0.56 |
| Variance Explained by PC1: 39.9% | |

Supplementary Table S 3: Pattern matrix from principal component analysis (PCA) with Promax rotation. Loadings are shown for three components identified as PC1: Verbal Fluency and Associative Memory, PC2: Processing Speed and Cognitive Flexibility, and PC3: Inhibition and Naming Speed. Values represent rotated factor loadings. Bold values indicate primary loadings (>0.4) on each component. The three components explained a cumulative variance of 63.9%.

| Test | **PC 1: Verbal Fluency and Associative Memory** | **PC 2: Executive Speed** | **PC 3: Inhibition and Naming Speed** |
| --- | --- | --- | --- |
| Face Name Test total | **0.56** | 0.27 | -0.07 |
| TMT-A [s] | -0.15 | **0.85** | 0.12 |
| TMT-B [s] | -0.16 | **0.95** | -0.06 |
| Stroop A [s] | -0.12 | -0.02 | **0.84** |
| Stroop B [s] | 0.01 | 0.01 | **0.87** |
| Stroop C [s] | 0.27 | 0.05 | **0.59** |
| Letter fluency: F [correct items] | **0.54** | 0.36 | -0.06 |
| Letter fluency: A [correct items] | **0.63** | 0.25 | 0.00 |
| Category fluency: supermarket [correct items] | **0.78** | -0.15 | 0.03 |
| Category fluency: animals [correct items] | **0.96** | -0.37 | 0.00 |
| Rotation: Promax. Cumulative Variance Explained: 63.9% | | | |

Supplementary Table S 4: Regression model for demographic predictors of global cognitive composite scores after StepAIC forward & backward predictor selection.
Significance levels: * *p* < 0.05, ***p < 0.01, *** p < 0.001*.

|  | PC:Global Cognition | | | |
| --- | --- | --- | --- | --- |
| *Predictors* | *Estimates* | *CI* | *Statistic* | *p* |
| (Intercept) | -0.33 | -0.59 – -0.07 | -2.55 | **0.01** |
| Female Sex | 0.60 | 0.25 – 0.95 | 3.43 | **<0.01** |
| Age | -0.40 | -0.58 – -0.23 | -4.58 | **<0.01** |
| Observations | 98 | | | |
| R^2^ / R^2^ adjusted | 0.268 / 0.252 | | | |
| AIC | 254.595 | | | |

Supplementary Table S 5: Regression model for upper limb motor predictors of global cognitive composite scores after StepAIC forward & backward predictor selection.
Significance levels: * *p* < 0.05, ***p < 0.01, *** p < 0.001*.

|  | **PC:Global Cognition** | | | |
| --- | --- | --- | --- | --- |
| ***Predictors*** | *Estimates* | *CI* | *Statistic* | *p* |
| **(Intercept)** | 0.00 | -0.17 – 0.17 | 0.00 | 1.00 |
| **mean 9-HPT time** | -0.54 | -0.71 – -0.37 | -6.32 | **<0.01** |
| **Observations** | 98 | | | |
| **R^2^ / R^2^ adjusted** | 0.294 / 0.287 | | | |
| **AIC** | 249.008 | | | |

Supplementary Table S 6: Regression model for lower limb function and gait predictors of global cognitive composite scores after StepAIC forward & backward predictor selection.
Significance levels: * *p* < 0.05, ***p < 0.01, *** p < 0.001*.

|  | PC:Global Cognition | | | |
| --- | --- | --- | --- | --- |
| *Predictors* | *Estimates* | *CI* | *Statistic* | *p* |
| (Intercept) | 0.00 | -0.17 – 0.17 | 0.00 | 1.00 |
| Extended balance score | 0.17 | -0.03 – 0.37 | 1.70 | 0.09 |
| 4 m walk test[s] | -0.18 | -0.37 – 0.02 | -1.82 | 0.07 |
| 5-chair rises test[s] | -0.22 | -0.42 – -0.02 | -2.19 | **0.03** |
| Min toe clearance(cm) | 0.24 | 0.07 – 0.41 | 2.75 | **0.01** |
| CV Swing(%) | -0.14 | -0.32 – 0.04 | -1.53 | 0.13 |
| CV Step length(%) | -0.19 | -0.37 – -0.01 | -2.10 | **0.04** |
| Observations | 98 | | | |
| R^2^ / R^2^ adjusted | 0.349 / 0.306 | | | |
| AIC | 251.038 | | | |

Supplementary Table S 7: Multiple Regression Model with Sex Interaction Terms Predicting the Global Cognitive Score. The table displays standardized beta coefficients (β) with 95% confidence intervals [95% CI] and p-values from the follow-up interaction analysis.

|  | PC:Global Cognition | | | |
| --- | --- | --- | --- | --- |
| *Predictors* | *Estimates* | *CI* | *Statistic* | *p* |
| (Intercept) | -0.20 | -0.44 – 0.05 | -1.61 | 0.11 |
| Female Sex | 0.43 | 0.11 – 0.74 | 2.66 | **0.01** |
| Age | -0.08 | -0.31 – 0.15 | -0.70 | 0.49 |
| mean 9-HPT time | -0.43 | -0.71 – -0.16 | -3.16 | **<0.01** |
| 4 m walk test[s] | -0.24 | -0.46 – -0.02 | -2.18 | **0.03** |
| 5-chair rises Test[s] | -0.25 | -0.56 – 0.05 | -1.65 | 0.10 |
| Min toe clearance(cm) | 0.24 | 0.01 – 0.48 | 2.05 | **0.04** |
| Female Sex × Age | -0.28 | -0.60 – 0.04 | -1.76 | 0.08 |
| Female Sex × mean 9-HPT time | 0.23 | -0.12 – 0.57 | 1.29 | 0.20 |
| Female Sex × 4 m walk test [s] | 0.13 | -0.20 – 0.47 | 0.80 | 0.43 |
| Female Sex × 5-chair-rises test [s] | -0.06 | -0.44 – 0.31 | -0.34 | 0.73 |
| Female Sex × Min toe clearance (cm) | -0.09 | -0.40 – 0.22 | -0.55 | 0.59 |
| Observations | 98 | | | |
| R^2^ / R^2^ adjusted | 0.556 / 0.499 | | | |
| AIC | 223.630 | | | |

Supplementary Table S 8: Multivariate Multiple Regression Results with Sex Interaction Terms Predicting Cognitive Domain Scores. The table displays standardized beta coefficients (β) with their corresponding 95% confidence intervals [95% CI] from the final multivariate model. The model uses the final selected predictor set to predict the three data-driven cognitive domain scores. The final row shows the Adjusted R² value for each domain-specific model. Significance levels: * p < 0.05, ** p < 0.01, *** p < 0.001.

| **term** | **PC 1: Verbal Fluency and Associative Memory** | **PC 2: Executive Speed** | **PC 3: Inhibition and Naming Speed** |
| --- | --- | --- | --- |
| **(Intercept)** | -0.25 [-0.52, 0.03] | 0.03 [-0.26, 0.31] | -0.20 [-0.50, 0.10] |
| **Female Sex** | **0.54 [0.18, 0.90]**** | -0.04 [-0.41, 0.34] | 0.41 [0.02, 0.80]* |
| **Age** | -0.02 [-0.28, 0.24] | -0.20 [-0.46, 0.07] | -0.00 [-0.28, 0.28] |
| **mean 9-HPT time** | **-0.39 [-0.70, -0.09]*** | **-0.34 [-0.66, -0.02]*** | -0.28 [-0.62, 0.05] |
| **4 m walk test [s]** | -0.17 [-0.42, 0.08] | -0.19 [-0.45, 0.07] | -0.24 [-0.51, 0.03] |
| **5-chair rises Test [s]** | **-0.34 [-0.69, -0.00]*** | -0.09 [-0.44, 0.27] | -0.10 [-0.47, 0.28] |
| **Min toe clearance (cm)** | **0.28 [0.02, 0.55]*** | 0.15 [-0.13, 0.43] | 0.10 [-0.19, 0.39] |
| **Female Sex × Age** | -0.24 [-0.60, 0.12] | -0.23 [-0.60, 0.14] | -0.20 [-0.59, 0.19] |
| **Female Sex × mean 9-HPT time** | 0.29 [-0.10, 0.69] | 0.06 [-0.35, 0.46] | 0.13 [-0.30, 0.56] |
| **Female Sex × 4 m walk test [s]** | 0.04 [-0.34, 0.41] | 0.21 [-0.18, 0.60] | 0.11 [-0.29, 0.52] |
| **Female Sex × 5-chair-rises test [s]** | 0.06 [-0.36, 0.48] | -0.10 [-0.53, 0.34] | -0.19 [-0.65, 0.27] |
| **Female Sex × Min toe clearance (cm)** | -0.20 [-0.55, 0.15] | 0.07 [-0.29, 0.43] | -0.01 [-0.39, 0.37] |
| **Adjusted R²** | **0.36** | **0.31** | **0.24** |
